# Effects of Resistant Starch Interventions on Metabolic Biomarkers in Pre-Diabetes and Diabetes Adults

**DOI:** 10.3389/fnut.2021.793414

**Published:** 2022-01-12

**Authors:** Aswir Abd Rashed, Fatin Saparuddin, Devi-Nair Gunasegavan Rathi, Nur Najihah Mohd Nasir, Ezarul Faradianna Lokman

**Affiliations:** ^1^Nutrition Unit, Nutrition, Metabolism and Cardiovascular Research Centre (NMCRC), Institute for Medical Research, National Institutes of Health, Ministry of Health Malaysia, Setia Alam, Malaysia; ^2^Endocrine and Metabolic Unit, Nutrition, Metabolism and Cardiovascular Research Centre (NMCRC), Institute for Medical Research, National Institutes of Health, Ministry of Health Malaysia, Setia Alam, Malaysia

**Keywords:** resistant starch, type 2 diabetes, biomarkers, glucose, insulin

## Abstract

Simple lifestyle changes can prevent or delay the onset of type 2 diabetes mellitus (T2DM). In addition to maintaining a physically active way of life, the diet has become one of the bases in managing TD2M. Due to many studies linking the ability of resistant starch (RS) to a substantial role in enhancing the nutritional quality of food and disease prevention, the challenge of incorporating RS into the diet and increasing its intake remains. Therefore, we conducted this review to assess the potential benefits of RS on metabolic biomarkers in pre-diabetes and diabetes adults based on available intervention studies over the last decade. Based on the conducted review, we observed that RS intake correlates directly to minimize possible effects through different mechanisms for better control of pre-diabetic and diabetic conditions. In most studies, significant changes were evident in the postprandial glucose and insulin incremental area under the curve (iAUC). Comparative evaluation of RS consumption and control groups also showed differences with inflammatory markers such as TNF-α, IL-1β, MCP-1, and E-selectin. Only RS2 and RS3 were extensively investigated and widely reported among the five reported RS types. However, a proper comparison and conclusion are deemed inappropriate considering the variations observed with the study duration, sample size, subjects and their metabolic conditions, intervention doses, and the intervention base products. In conclusion, this result provides interesting insights into the potential use of RS as part of a sustainable diet in diabetes management and should be further explored in terms of the mechanism involved.

## Introduction

Diabetes mellitus is a metabolic disorder characterized by hyperglycemia due to defective insulin secretion, action or both (1). The diagnostic criteria for diabetes include fasting plasma glucose (FPG) ≥7.0 mmol/L (2), glycated hemoglobin (HbA1c) ≥6.5% (2–4), 2-h plasma glucose (2hPG) in a 75 g oral glucose tolerance test (OGTT) ≥11.1 mmol/L (2) or random PG ≥11.1 mmol/L. Meanwhile, pre-diabetes refers to impaired fasting glucose (IFG) of 6.1–6.9 mmol/L (5), impaired glucose tolerance (IGT)/2hPG in a 75 g OGTT of 7.8–11.0 mmol/L (5) or HbA1c of 6.0% to 6.4% (6), each of which places individuals at high risk of developing diabetes and its complications (7).

A recent study by Khan and his team (8) presented the epidemiology of type 2 diabetes mellitus (T2DM) in terms of the global burden of disease and forecasted trends. The study showed that T2DM continues to be the leading cause of human suffering and deaths as it continues to increase in prevalence and incidence. This phenomenon continues, and there are no sights for reduction despite efforts in clinical care, research and public health interventions (8). Globally, it was recognized that diets low in whole grains, nuts, seeds and fruits were the leading dietary risks, especially in developing countries where globalization and emerging supermarkets increase access to processed, salt-laden, high-fat and sugar-added food products. Consequently, the attractiveness in line with low prices and higher accessibility led to decreased intake of whole grains, fruits and vegetables. In addition, poor dietary control and a sedentary lifestyle contribute to higher BMI that further elevates the associated risks (9).

The nutritional recommendation based on FAO/WHO states that the acceptable macronutrient distribution range should be within 55–75% of net energy for carbohydrates, 10% for sugar components, while dietary fiber intake requirements differ with 38 g and 25 g for men and women, respectively (10). Several published findings have highlighted the association of high fiber and grains intake toward lower risk of both obesity and diabetes. However, the usual dietary pattern does not meet the recommended quantity, and hence it was suggested to include an additional grain-fibrous food for a more enhanced approach in managing metabolic conditions, such as diabetes (11–13).

**Figure 1:**
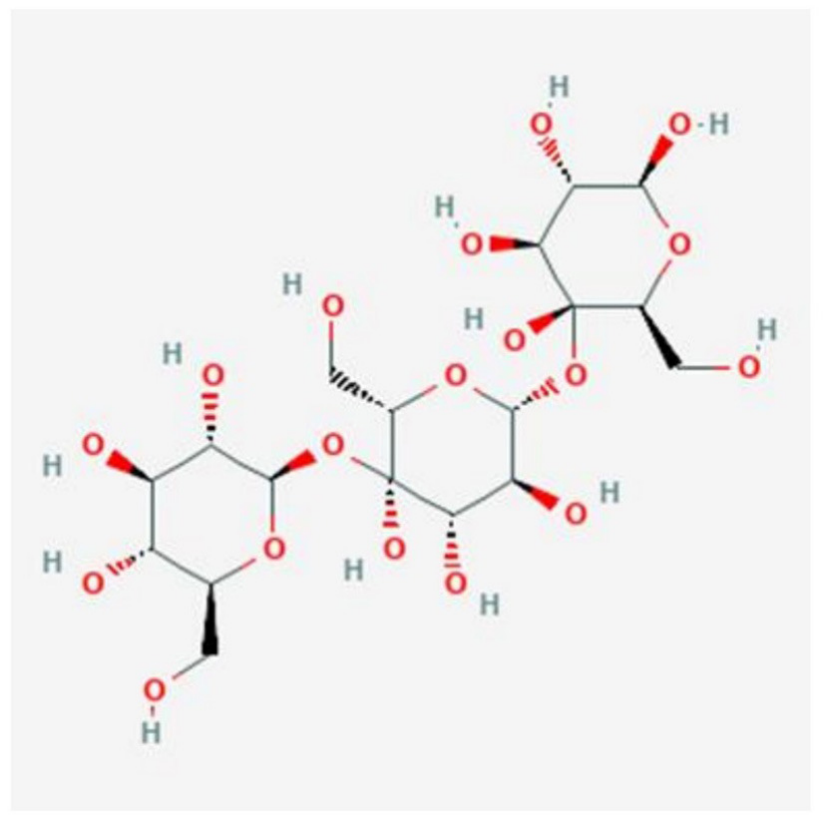
Chemical structure of α-1,4-D-glucan [Obtained from: (17)].

Starch is the predominant form of carbohydrate and is categorized as rapidly digestible starch (RDS), slowly digestible starch (SDS) and resistant starch (RS) determined based on their digestion rate. SDS seems to be slowly digested within the small intestines, whereas RDS tends to elevate glucose levels rapidly in the blood upon consumption. On the other hand, RS is undigested in the small intestines and usually fermented in the colon (14). Several studies have shown that RS is a linear molecule of α-1,4-D-glucan, derived from the retrograded amylose fraction and has a relatively low molecular weight (1.2 × 10^5^ Da) (15, 16). The chemical structure of α-1,4-D-glucan is shown in Figure 1 (17).

RS is used widely as a functional ingredient, especially in foods containing high dietary fiber levels. These types of food are assumed to help prevent several pathologies such as colon cancer, diabetes, or obesity (18). It has been proposed that foods containing naturally occurring RS or foods modified to contain more RS may alter the postprandial glycemic response, prevent hypoglycemia, reduce hyperglycemia and explain differences in some foods' glycemic index (GI). Dietary supplementation of RS is one of the nutritional interventions practiced for metabolic disease (19). Bread, cooked cereals and pasta, vegetables, just-ripe bananas are several familiar food products that serve as natural as natural RS sources. Consumption of RS foods tends to resist digestion in the small intestines and reaches the large intestines for microbial fermentation (14, 20, 21).

There are five types of RS based on their indigestible properties (RS1, RS2, RS3, RS4, and RS5) (16, 17). RS1 refers to physically entrapped starch within whole plant cells and food matrices (e.g., partly milled grains and seeds) where there is a physical barrier to amylolysis. The presence of intact cell walls contributes to the RS content of legumes. Extensive milling (and chewing) allows these starches to be more accessible and less resistant. RS2 comprises the poorly gelatinized and hydrolyzed granules by α-amylases from certain plants (e.g., raw potato and green banana, HAM). Retrograded starches constitute RS3, which includes cooked and cooled rice or potatoes. Meanwhile, RS4 is a group of chemically modified starches that can improve the functional characteristics of the starch. Amylose-lipid complex has been proposed as RS5 because high-amylose starch tends to be more resistant to enzyme hydrolysis than low-amylose starch. The amylose-lipid complex in starch granules increases their enzyme resistance by restricting the granule swelling during cooking. Although these modified starches are found widely in processed foods, neither their contribution to RS intakes nor their physiological effects have been extensively studied (22).

RS has the advantage of having a less negative influence on the sensory properties of final products than traditional fibers, such as whole grains, bran or fruit fibers, which is very positive for consumer acceptability (23). Consumption of RS has resulted in changes in insulin sensitivity, IGT and satiety in healthy humans (24) and therefore has been hypothesized to have implications for glycemic control in individuals at risk of or with T2DM. Among the five types, RS2 and RS3 were the most common form utilized for interventions, especially in evaluating their effects on blood lipids, GI and colon cancer (24, 25). RS-based diets are one of the nutritional interventions practiced for metabolic disease control, and these include common food sources such as bread, cooked cereals and pasta, vegetables and just-ripe bananas. Despite the numerous interventions on evaluating RS effects on diabetic control, the findings seem to be limited to short-term studies and lack longer-term studies to prove the benefits of RS. Thus, in this review, we intend to explore the reported studies on RS diet interventions to further understand its importance as well as to investigate the possible effects exerted by various RS-based intervention studies among pre-diabetes and diabetes adults.

## Materials and Methods

### Search Strategy

**Figure 2:**
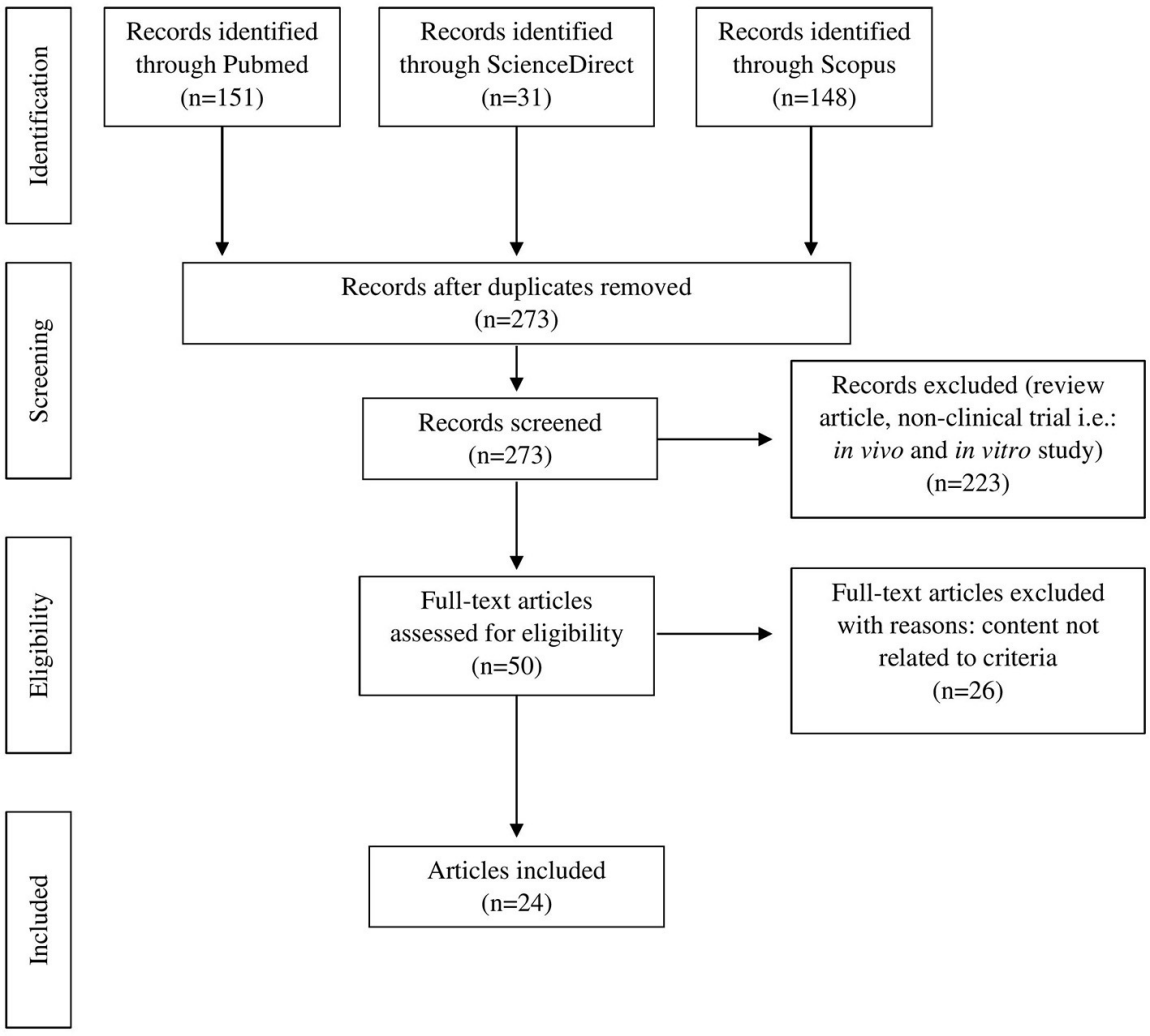
PRISMA flow diagram of literature search and selection process.

Original articles were searched in three databases (PubMed, Scopus and ScienceDirect) from the year 2011 to 2021 using the Medical subject heading (MeSH) terms “diabetes,” crossed with the term “resistant starch.” Publications with available abstracts were reviewed and limited to studies published in English. Papers on human and clinical trials related to diabetes were included. However, review articles, proceedings, letters to the editor, and *in vitro* and *in vivo* studies were excluded. Duplicate articles were eliminated. The study identification process and reasons for exclusion are illustrated in Figure 2.

### Eligibility Criteria

We included published intervention studies (defined as a randomized controlled trial, crossover study and quasi-experimental study) comparing markers of glucose metabolism for RS consumption. We included only human studies with adult participants aged at least 18 years old from both genders. Studies were included if they analyzed at least one of the biomarkers as mentioned earlier.

### Study Selection

A pair of authors independently assessed the titles and abstracts during the initial screening. The difference in the initial assessment was resolved by a discussion leading to a consensus, with a third party serving as arbitrator if necessary. Each study was recorded as include, exclude or unclear. Full articles were retrieved for further assessment if recorded as include or unclear. Any disagreement was re-evaluated and re-assessed among the reviewers.

## Results

The search resulted in 50 articles produced with a refined search based on the availability of full text, peer-reviewed articles and library collection access. Upon further assessment, only 24 full-text articles were relevant and included for final review (Table 1). All the related articles were printed out for further assessment of evidence-based to explore the effectiveness of RS as a potential antidiabetic agent.

**Table 1:** The potential health benefit effect of RS interventions on diabetic biomarkers.

**References**	**Objective (s)**	**Methods**	**Findings**	**Conclusion**
Bodinham et al. (26)	To further explore the effects of RS on insulin secretion.	• A subject-blind, randomized crossover study. • 12 overweight individuals (37 ± 4.0 yrs) consumed either 40 g RS2 or energy and carbohydrate (CHO)-matched placebo daily, for 4 wks. • Assessment of insulin secretion, plasma insulin and C-peptide concentrations.	• Significantly higher plasma insulin and C-peptide concentrations with RS (*p* < 0.05). • Significantly improved 1st phase insulin secretion with RS (*p* < 0.05). • No changes on body weight or habitual food intake.	• RS intake significantly increased the 1st -phase insulin secretion in individuals at risk of developing T2DM. • Further studies exploring this effect in individuals with T2DM are required.
Bodinham et al. (27)	To determine the effects of increased RS consumption on insulin sensitivity and glucose control and changes in postprandial metabolites and body fat in T2DM.	• A single-blind, randomized dietary intervention crossover study. • 17 individuals (mean age 55 yrs) with well-controlled T2DM consumed either 40 g of HAM-RS2 or placebo for 12 wks. • Three metabolic investigations: a two-step euglycemic–hyperinsulinemic clamp combined with an infusion of [6,6-^2^H_2_] glucose, a meal tolerance test (MTT) with arterio-venous sampling across the forearm, and whole-body imaging. • Determination of plasma glucose, insulin, triglycerides (TG), non-esterified fatty acids (NEFA), total cholesterol (TC) and high-density lipoprotein (HDL). • Determination of fasting tumor necrosis factor-α (TNF-α) and interleukin (IL) 6, C-peptide and total glucagon-like peptide-1 (GLP1).	• Significantly lower postprandial glucose concentrations (*p* = 0.045). • No effect of HAM-RS2 on hepatic, peripheral insulin sensitivity, or HbA1c. • No significant difference in C-peptide, HOMA, TC, HDL and IL6 between the HAM-RS2 and placebo. • Significant difference in NEFA, TG and TNF-α. • Fasting GLP1 concentrations were significantly lower following HAM-RS2 consumption (*p* = 0.049). • Significantly greater postprandial GLP1 excursions during the MTT (*p* = 0.009).	• HAM-RS2 did not improve tissue insulin sensitivity in well-controlled T2DM, but demonstrated beneficial effects on meal handling, possibly due to higher postprandial GLP1.
Dainty et al. (28)	To examine the chronic effects of consuming bagels high in HAM-RS2 on fasting and postprandial glycemic markers in adults at increased risk of T2DM.	• A randomized, double-blind crossover design. • 24 men and women (mean age of 55.3 ± 1.59 yrs and body mass index (BMI) of 30.2 ± 0.57 kg/m^2^) consumed 1 bagel containing 25 g/d HAM-RS2 or one control wheat bagel for 56 d each, separated by a 4 wks washout. • Fasting and postprandial OGTT glucose and insulin.	• Significantly lower fasting, 2 h and 3 h insulin incremental area under the curve (iAUC) and fasting insulin resistance (IR) than control (*p* < 0.05). • No difference in fasting and postprandial OGTT glucose concentrations.	• Consumption of a high-HAM-RS2 bagel improves glycemic efficiency and fasting insulin sensitivity in adults at increased risk of T2DM.
Peterson et al. (29)	To test whether RS2 can improve cardiometabolic health among pre-diabetic adults.	• A randomized, double-blind, placebo-controlled, parallel-arm trial. • 68 overweight adults (35–75 yrs) with pre-diabetes were randomized to consume 45 g/d of HAM-RS2 or an isocaloric amount of the RDS amylopectin (control) for 12 wks. • HbA1c, insulin sensitivity, insulin secretion, ectopic fat, and markers of inflammation.	• RS2 lowered HbA1c by a clinically insignificant (*p* > 0.05). • RS2 also did not affect insulin sensitivity, TG, TC, FFA, high-sensitive (hs)-CRP, iAUC relative to baseline (*p* > 0.05). • Significant reduction in TNF-α, and heart rate (*p* < 0.05).	• RS supplementation reduced the inflammatory marker TNF-α and heart rate, but it did not significantly improve glycemic control and other cardiovascular disease risk factors among pre-diabetic adults.
Kwak et al. (30)	To evaluate whether 4 wks of dietary treatment with rice containing RS reduces blood glucose and oxidative stress as well as improves endothelial function.	• Patients with IFG, IGT or newly diagnosed T2DM (*n* = 90) were randomly assigned to either rice containing 6.51 g RS/d or a control rice group for 4 wks. • Fasting and postprandial levels of glucose and insulin, oxidative stress markers and endothelial function.	• Significant reduction on fasting insulin and IR, postprandial glucose (*p* < 0.010) and insulin levels at 30 min, and glucose and iAUC after the standard meal. • Decreased urinary 8-epi-PGF_2α_ and plasma malondialdehyde (MDA) and increased the RH-PAT index (*p* < 0.001) and total nitric oxide (NO). • Postprandial changes in glucose at 60 and 120 min and areas under the glucose response curve, MDA, RH-PAT, and total NO of the test group differed significantly from control.	• In patients with IFG, IGT or newly diagnosed T2DM, rice containing RS was associated with improved endothelial function reduction of postprandial glucose and oxidative stress compared with control.
Lotfollahi et al. (31)	To investigate the effects of 6 mths consumption of green-banana biomass on the LDL particle functionality in subjects with T2DM.	• Subjects (*n* = 39, mean age 65 yrs) of both sexes with diabetes (HbA1c ≥ 6.5%) were randomized to receive nutritional support plus green-banana biomass (40 g) (*n* = 21) or diet alone (*n* = 18) for 6 months. • Non-linear optical responses of LDL solutions from these participants were studied by Z-scan technique. • Measurement absorbance structural changes in LDL samples and determination of LDL sub-fractions.	• Significant reduction on total- and non-HDL-cholesterol, glucose, HbA1c and improved the protection of the LDL particle against oxidation, by increasing carotenoids content in the particles (*p* < 0.05).	• Higher protection against modifications may decrease the risk of developing cardiovascular disease. • Benefits of the green-banana biomass encourage the RS usage with potential clinical applications among pre-diabetic and diabetic individuals.
Gargari et al. (32)	To determine effects of RS2 on metabolic parameters and inflammation in women with T2DM.	• A randomized controlled clinical trial. • 60 females (30–65 yrs) with T2DMreceived 10 g/d RS2 or placebo for 8 wks. • FPG, HbA1c, lipid profile, hs-CRP, IL-6 and TNF-α.	• RS2 significantly decreased HbA1c (−0.3%, −3.6%), TNF-α (−3.4 pg/mL, −18.9%) compared with placebo (*p* < 0.05). • Changes in FBS, hs-CRP and IL-6 were not significant.	• RS2 can improve glycemic status, inflammatory markers and lipid profile in women with T2DM. • More studies are needed to confirm efficacy of RS2 as an adjunct therapy in diabetes.
Alfa et al. (33)	To determine the tolerability as well as the glucose and insulin modulating ability of *MSPrebiotic*^®^ digestion RS in healthy mid-age (MID) and elderly (ELD) adults.	• A prospective, blinded, placebo-controlled study. • ELD (>70 yrs) and MID (30–50 yrs) consumed either 30 g/d *MSPrebiotic*^®^ or placebo for 12 wks. • Blood glucose, lipid profile, C-reactive protein (CRP), lipid particles, TNF-α, IL-10, insulin and IR.	• A significant difference in blood glucose (*p* = 0.0301) and insulin levels (*p* = 0.009), as well as IR (HOMA-IR; *p* = 0.009) in ELD adults who consumed *MSPrebiotic^®^*. • *MSPrebiotic*^®^ consumption for 12 wks was not sufficient to reduce the elevated CRP and TNF-α levels in the ELD group. • No significant changes in MID adults.	• Dietary supplementation with prebiotics such as *MSPrebiotic*^®^ may be part of an effective strategy to reduce IR, in the ELD.
Giles et al. (34)	To determine the *in vivo* net energy content of RS and examine its effect on macronutrient oxidation.	• A randomized, double-blind cross-over study. • 18 healthy adults aged 25–45 yrs. • Measurement of total energy expenditure (TEE), substrate oxidation, and postprandial metabolites in response to three diets: (a) digestible starch (DS), (b) RS (33% dietary fiber), (c) RS with high fiber (RSF, 56% fiber).	• The in vivo net energy content of RS and RSF are 2.74 ± 0.41 and 3.16 ± 0.27 kcal/g, respectively. • No difference in TEE or protein oxidation between DS, RS, and RSF. • RS and RSF consumption caused a 32% increase in fat oxidation (*p* = 0.04) with a concomitant 18% decrease in CHO oxidation (*p* = 0.03) vs. DS. • Insulin responses were unaltered after breakfast but lower in RS and RSF after lunch, at equivalent glucose concentrations.	• RS and RSF consumption increase fat and decrease CHO oxidation with postprandial insulin responses lowered after lunch.
Belobrajdic et al. (35)	To determine if bread made from HAW and enriched in RS dampens postprandial glycemia compared with bread made from conventional low-amylose wheat (LAW).	• A single-center, randomized, double-blinded, crossover- controlled study. • 20 healthy non-diabetic men and women (mean age 30 ± 3 yrs; BMI 23 ± 0.7 kg/m^2^) consumed a glucose beverage or 4 different breads (LAW-R (refined), LAW-W (wholemeal), HAW-R, or HAW-W) for 7 wks. • Plasma glucose, insulin, ghrelin, incretin hormone concentrations and iAUC.	• HAW breads: iAUC: 39% < conventional wheat breads (HAW 39 ± 5 mmol/L × 3 h; LAW 64 ± 5 mmol/L × 3 h; *p* < 0.0001). • Insulinemic and incretin: 24–30% less for HAW breads than for LAW breads (p < 0.05). • Flour processing did not affect the glycemic, insulinemic, or incretin response. • The HAW breads did not influence plasma ghrelin.	• Replacing LAW with HAW flour may be an effective strategy for lowering postprandial glycemic and insulinemic responses to bread in healthy men and women, but further research is warranted.
Hallström et al. (36)	To evaluate the postprandial glucose and insulin responses *in vivo* to bread products based on a novel wheat genotype with elevated amylose content (EAW) of 38%.	• A randomized cross-over trial. • Healthy 7 females and 7 males (20–35 yrs; BMI: 22.2 ± 1.91) were served test meals on 4 occasions. • RS content (*in vitro*), postprandial glucose and insulin responses.	• Significantly higher RS content in EAW bread than in whole grain wheat bread (WGW) (*p* < 0.001). • EAW induced lower postprandial glucose response than white wheat flour (REF) during the first 120 min (*p* < 0.05), but no significant differences in insulin responses. • Increased RS content per test portion was correlated to a reduced GI (*r* = −0.571, *p* < 0.001).	• Wheat with EAW may be preferable to other wheat genotypes considering RS formation, however further research is required.
Poquette et al. (37)	To measure the contents of functional starch fractions, SDS and RS, and to investigate the effects of grain sorghum on postprandial plasma glucose and insulin levels.	• A randomized-crossover design. • 10 healthy males (25.1 ± 4 yrs) consumed grain sorghum and whole wheat flour (control) muffins containing 50 g total starch with a 1 wk washout period. • Measurement of glucose and insulin levels at 15 min before and 0, 15, 30, 45, 60, 75, 90, 120, 180 min after consumption.	• Mean glucose and insulin responses reduced at 45–120 min and 15†90 min with grain sorghum, compared to control (*p* < 0.05). • The iAUC was significantly lowered for plasma glucose responses (*p* < 0.05). • Significant reduction with insulin responses with sorghum (*p* < 0.05).	• Grain sorghum is a good functional ingredient to assist in managing glucose and insulin levels in healthy individuals.
Gu (38)	To investigate the effects of sorghum starch on postprandial blood glucose and insulin levels in pre-diabetic men	• Grain sorghum and wheat (control) muffins containing 50 g total starch were consumed by 15 pre-diabetic males on two mornings with a 1 wk washout period. • Measurement of glucose and insulin levels at −15 (baseline), 0, 15, 30, 45, 60, 75, 90, 120, and 180 min after each treatment.	• The functional starch content [combined SDS and RS] of grain sorghum muffin was higher than control. • Postprandial blood glucose and insulin responses were both significantly reduced at 45–120 min intervals (*p* < 0.05). • The mean iAUC of glucose and insulin was significantly reduced by 35 and 36.7%, respectively (*p* < 0.05).	• Grain sorghum is a good candidate in controlling blood glucose and insulin levels in pre-diabetic population for the prevention of T2DM.
Lin et al. (39)	To evaluate the effects of the new RS formula, PPB-R-203, on glucose homeostasis in healthy subjects and subjects with T2DM.	• A cohort consisting of 40 healthy participants (20–65 yrs) received test and control diets. • A randomized, 2-regimen, cross-over, comparative study was conducted in 44 subjects (20–65 yrs) with T2DM and glycemic control was assessed with a continuous glucose monitoring system. • Determination of blood glucose and iAUC	• Serum glucose values and iAUC were significantly lower in the PPB-R-203 than the control group, for healthy subjects (*p* < 0.05). • In patients with T2DM, mean blood glucose concentrations for control regimen were higher than the PPB-R-203-based regimen (*p* = 0.023). • AUCs for total blood glucose and hyperglycemia were also reduced for subjects on the PPB-R-203- compared to control (total blood glucose: *p* < 0.001; hyperglycemia: *p* = 0.021).	• A PPB-R-203-based diet reduced postprandial hyperglycemia in patients with T2DM without increasing the risk of hypoglycemia or glucose excursion.
Sanders et al. (40)	To evaluate the effect of consuming cooked, then chilled potatoes, compared to isoenergetic, CHO-containing control foods.	• A pilot cross-over randomized controlled trial. • 19 adults (18–74 yrs;BMI 27.0–39.9)consumed 300 g/day RS-enriched potatoes, over a 24 h period. • Assessment of insulin sensitivity, fasting plasma glucose and fasting insulin	• No significant difference for insulin sensitivity between potato and control. • Lower fasting plasma glucose (*p* = 0.043) with potato compared to control. • Lower fasting insulin (*p* = 0.077) in the potato vs. control.	• RS-enriched potatoes may have a favorable impact on CHO metabolism and support the view that additional research in a larger study sample is warranted.
Mohan et al. (41)	To compare the GI of a newly developed high fiber white rice (HFWR) with that of commercial white rice (WR)	• A randomized controlled crossover study design. • 30 healthy adults age 18–45 yrs were recruited for the GI study of HFWR in 2013 • In 2014, GI testing of the second harvest HFWR was done in a subsample of 15 healthy volunteers. • HFWR and WR diets providing 50 g of available CHO (63.6 g of uncooked rice) were given as test foods	• Dietary fiber content of HFWR was 5-fold higher. • RS content of HFWR was 6.5-fold higher (*p* < 0.001) • Amylose content of HFWR was significantly higher (*p* < 0.001) compared with WR • HFWR had 23% lower GI compared with WR (*p* = 0.002).	• HFWR has lower GI excellent sensory and other characteristics compared with WR. • Switching from the current high GI WR to HFWR could help to reduce overall dietary GI and the glycemic load.
Nomura et al. (42)	To evaluate the postprandial glycemic response for boiled BARLEYmax^®^ and determined its GI in a Japanese population.	• 11 healthy subjects (20–50 yrs) were administered a 50 g/150 mL glucose drink twice and boiled BARLEYmax^®^ containing 50 g available CHO after a wash-out period. • Determination of blood glucose, postprandial blood glucose, glucose iAUC and GI of BARLEYmax^®^.	• Postprandial blood glucose, its change from baseline over 90 min, and the iAUC for BARLEYmax^®^ were statistically lower than those for the glucose drink. • The GI of the BARLEYmax^®^ was 24.3.	• Boiled BARLEYmax^®^ contributes to improving the postprandial glycemic response.
Zhu et al. (43)	To examine the possibility of integrating domestically cooked non-cereal starchy foods into glycemic management diet, and compare their glycemic characteristics with those of waxy and non-waxy whole grains and starchy beans.	• An undouble-blind, randomized crossover design. • 10 healthy subjects (18–26 yrs) consumed dried lily bulb (LB), lotus seed (LS), adlay (AD), waxy black rice (BR), millet (MI), and adzuki bean (AB), pre-soaked. • *In vitro* CHO digestion for each test food.	• Both the LS and AB meals achieved low GI (21–51), while the other starchy foods failed to show significant difference with rice GI (83–109). • The hydrolysis indexes of LS and AB were 37.7–61.1%, significantly lower than other test foods. • The *in vitro* tests indicated that pre-soaking resulted in high RDS and low RS.	• Careful choice of whole grain materials, minimized pre-soaking, and moderate cooking may be critical factors for successful postprandial glycemic management for diabetic and pre-diabetic.
Yulianto et al. (44)	To evaluate content of RS, and GI of Cr—fortified—parboiled rice (Cr-PR) coated with herbal extracts.	• 18 non-diabetic volunteers were recruited to test on GI of the cooked Cr-PR coated with herbal extract. • Unhulled rice and forticant used (Ciherang and CrCl3). Three herbal extracts used were cinnamon bark powder, pandan leaf and bay leaf. • Determination of RS content by enzymatic process.	• RS content of Cr-PR coated with herbal extracts ranged between 8.27 and 8.84%. • Cr-PR coated with herbal extract of 3% had higher RS levels than herbal extracts of 6% and 9% (*p* < 0.05). • Rice coated with 3% cinnamon extract showed the highest RS content (8.84%). • The lowest GI (29–30) was attained by the Cr-PR coated with cinnamon extract of 6–9%.	• The low GI of Cr-PR may be more influenced by the potential of polyphenolic compounds in the herbal extract than its RS levels.
Sandberg et al. (45)	To investigate the effect of WG rye-based products on glucose- and appetite regulation.	• A crossover overnight study design. • 21 healthy subjects (25.3 ± 3.9 yrs) were provided four rye-based evening test meals of either WG rye flour bread (RFB) or a 1:1 ratio of WG rye flour and rye kernels bread (RFB/RKB), with or without added RS. • Determination of blood glucose, insulin, peptide YY (PYY), FFA, IL-6, *ad libitum* energy intake as well as breath H_2_ and subjective rating of appetite.	• The evening meal with RFB/RKB + RS decreased postprandial glucose- and iAUC (*p* < 0.05). • All rye-based evening meals decreased or tended to decrease fasting FFA (*p* < 0.05, RFB/RKB: *p* = 0.057). • The evening meal comprising RFB/RKB + RS resulted in an increased p-PYY concentration at fasting (+17%, *p* < 0.05). • No effects on energy intake or IL-6 compared to WWB. • All rye-based evening meals resulted in increased breath H_2_ levels at fasting, that remained increased after the standardized breakfast (*p* < 0.001).	• WG rye bread has the potential to improve cardiometabolic variables in an 11–14.5 h perspective in healthy humans. • The combination RFB/RKB + RS positively affected biomarkers of glucose- and appetite regulation in a semi-acute perspective. Meanwhile, RFB and RFB/RKB improved subjective appetite ratings.
Sandberg et al. (46)	To investigate the effects of short-term intervention with WG rye on cognitive functions, mood and cardiometabolic risk markers in MID test subjects.	• Crossover study • 38 healthy MID subjects consumed rye-based breads made up of WG rye kernel/flour (1:1) supplemented with RS2 (RB + RS2) for three consecutive days, with white wheat flour bread as reference. • Cognitive function, mood and cardiometabolic risk markers were determined the following morning, 11 – 14 h post intake.	• RB + RS2 increased insulin sensitivity (*p* < 0.05), (PYY, *p* < 0.05; GLP-2, *p* < 0.01) and fasting concentrations of plasma acetate, butyrate and total SCFA (*p* < 0.001). • Fasting levels of IL – 1β were decreased (*p* < 0.05). • No significant difference for other inflammatory markers (CRP, IL-6, IL-18 and LBP) and blood lipids (FFA and TG). • Insulin sensitivity was positively correlated with working memory test performance (*p* < 0.05).	• This study displays novel findings regarding effects of WG rye products on mood, and glucose and appetite regulation in MID subjects, indicating anti-diabetic properties of WG rye. • The beneficial effects are suggested to be mediated through gut fermentation of dietary fiber in the RB + RS2 product.
Nichenametla et al. (47)	To examine the effects of a blinded exchange of RS4-enriched flour (30% v/v) with regular/control flour (CF) diet on multiple metabolic syndrome (MetS) comorbidities.	• A double blind, placebo-controlled, cluster cross-over intervention. • 86 male and female subjects (≥ 18 yrs) consumed RS4-enriched flour (30% v/v) and regular flour as control with 2 wks washout period • Determination of glucose profile (FPG, post-prandial glucose, and HbA1C).	• RS4 consumption resulted in 7.2% (*p* = 0.002) lower mean TC, 5.5% (*p* = 0.04) lower non-HDL, and a 12.8% (*p* < 0.001) lower HDL in the MetS group. • No significant effect of RS4 was observed for glycemic variables (FPG, postprandial glucose, and HbA1C) and blood pressures.	• RS4 consumption improved dyslipidemia and body composition.
García et al. (48)	To evaluate the glycemic control and cardiovascular risk biomarkers in fragile, ELD T2DM patients after the intake of a new fructose-free diabetes-specific formula enriched with RS4 and high in monounsaturated FAs.	• An experimental, prospective, intention-to-treat clinical trial. • 41 patients with T2DM (78.9 ± 2.8 yrs) were fed exclusively with an enteral diabetes-specific formula for 6 wks. • Data were collected at baseline and after 6 wks of feeding. • CHO and lipid metabolism, inflammatory and cardiovascular risk biomarkers were measured.	• Blood HbA1c significantly decreased after the intervention (*p* < 0.05), as well as monocyte chemotactic protein-1 (MCP-1) and soluble E-selectin (*p* < 0.05). • Soluble vascular cell adhesion molecule-1 (sVCAM-1) and plasminogen activator inhibitor-1 (PAI-1) tended to decrease from baseline to 6 wks (*p* = 0.084 and *p* = 0.05, respectively).	• The new product improves glycemic control and cardiovascular risk without altering lipid metabolism, which is useful for the prevention of diabetic complications. • Longer intervention studies are needed.
Rahat-Rozenbloom et al. (49)	To compare the effects of two fermentable fibers on postprandial SCFA and second-meal glycemic response in healthy overweight or obese (OWO) vs. lean (LN) participants.	• Randomized crossover design. • Male and non-pregnant, non-lactating females aged 18–65 yrs. • 13 OWO and 12 LN overnight fasted participants were studied for 6 h on three separate days after consuming 300 mL water containing 75 g glucose as control or with 24 g IN or 28 g RS. • Determination of blood and serum glucose, insulin, C-peptide and FFA.	• IN significantly increased serum SCFA (*p* < 0.001) but had no effect on FFA or second-meal glucose and insulin responses compared to control. • RS had no significant effect on SCFA but reduced FFA rebound (*p* < 0.001) and second-meal glucose (*p* = 0.002) and insulin responses (*p* = 0.024). • OWO had similar postprandial serum SCFA and glucose concentrations but significantly greater insulin and FFA than LN. • The effects of IN and RS on SCFA, glucose, insulin and FFA responses were similar in LN and OWO.	• RS has favorable second-meal effects, likely related to changes in FFA rather than SCFA concentrations. However, a longer study may be needed to demonstrate RS effects on SCFA. • No evidence that acute increases in SCFA after IN reduce glycemic responses. • No significant differences detected in SCFA responses between OWO vs. LN subjects.

## Discussion

Table 1 was constructed based on available intervention studies on RS over the last decade. Although there are five types of RS, only three types have been clinically studied based on our search strategies from the three selected databases. HAM-RS2, an ingredient available to both food producers and consumers, was the usual form of RS2 used. HAM-RS2 is a bland, white substance with a small particle size comprised of 60% RS and 40% SDS (50). A randomized crossover study done by Bodinham and her colleagues on 12 overweight individuals reported significantly higher plasma insulin and C-peptide concentrations in individuals who consumed 40 g/d RS2 for 4 wks compared with the placebo (27). The process of insulin synthesis involves cleaving stages of C-peptide from proinsulin and is stored within secretory granules before its release in equimolar amounts with insulin into the bloodstream. C-peptide plays a vital role during this process by linking the A and B chains leading to precise folding and formation of interchain disulphide bonds (51). The presence of a higher level of both insulin and C-peptide assists the cells for better glucose absorption, blood sugar reduction and channeling glucose to the cells for energy synthesis. It is important to note that the circulating venous (or arterial) fasting insulin concentrations are about 18–90 pmol/L in healthy LN individuals (52). However, no changes were reported to either bodyweight or habitual food intake of the subjects in the study by Bodinham and team (26).

In another crossover study done by the same group of researchers on individuals with well-controlled T2DM, RS2 consumption resulted in significantly lower postprandial glucose concentrations (*p* < 0.05) without any effect on hepatic, peripheral insulin sensitivity or HbA1c levels (27). Several biochemical abnormalities were observed in insulin and glucagon secretion, uptake and suppression of hepatic glucose production, and uptake of peripheral glucose among diabetic individuals that led to higher and prolonged postprandial glucose (PPG) excursions when compared with non-diabetic individuals (53). Generally, PPG accounted for ~80% of HbA1c when HbA1c was <6.2% and only about 40% when HbA1c was above 9.0% (54). Decreases in PPG accounted for nearly twice as much for the reduction in HbA1c as did the decline in FPG. HbA1c values reflect overall glycemic exposure over the past 2-3 mths integrating both FPG and PPG levels.

Despite no improvement effect of HAM-RS2 on tissue insulin sensitivity in well-controlled T2DM, it demonstrated beneficial effects on meal handling, possibly due to higher postprandial glucagon-like peptide-1 (GLP1). GLP-1 delays gastric emptying and gut motility in healthy LN and obese subjects and patients with T2DM (55, 56). GLP-1 also contributes to the change in gastric volume that occurs in anticipation of food ingestion (57). In contrast, a double-blind crossover study done by Dainty and her colleagues in adults at an increased risk of T2DM characterized by a Canadian diabetes risk assessment questionnaire (CANRISK) score ≥21 (58) found that consumption of one bagel containing 25 g/d HAM-RS2 for 8 wks did not show any differences in FPG and PPG between the RS-intervention and control. However, the HAM-RS2 bagel recorded significantly lower fasting insulin iAUC and fasting IR (28). These findings are consistent with the results of another study done by Galarregui and co-workers that found positive associations of both fasting insulin and HOMA-IR with the insulin iAUC for foods with varied macronutrient composition consumed by senior participants (59). Interestingly, an investigation by Peterson and co-investigators (29) also recorded no improvement in glycemic control, cardiovascular disease risk factors and energy metabolism relative to baseline when pre-diabetic adults consumed HAM-RS2 for 12 wks. The sole exception was a decrease in circulating concentrations of TNF-α.

A possible explanation for the inconsistent findings of RS2 is partly because of the underlying dietary variability among individuals who participated in the studies. As mentioned earlier, RS2 supplementation did not improve cardiometabolic health in adults with pre-diabetes, although it does reduce TNF-α concentrations (29). Thus, it may be necessary to search for any relevant articles that correlate various factors in terms of gut microbiota, diet composition, biological and environmental factors that respond better to RS2 supplementation than others. For example, the possible mechanism in the management of T2DM via activation of protein phosphatase-4, TNF-α and plasminogen-activator inhibitors were reported in several studies (60, 61). These articles could lead to a better understanding of the potential beneficial effects of RS supplementation on metabolic health and whether such effects are modulated by diet compositions or existing microbial populations in the gut.

In addition, increasing evidence relates the roles of gut microbiota with T2DM development (62). Diabetic developments are also affected by chemical and diet-related factors (63). Recently, RS2 in the form of green-banana biomass studied in 39 T2DM patients of both genders for 6 mths showed significantly lower TC, non-HDL-cholesterol, glucose, and HbA1c levels (*p* < 0.05), as well as improved the LDL particles protection against oxidation which indirectly assists in lowering cardiovascular disease risk among T2DM patients (31). Gargari and co-investigators (32) have conducted a study to determine if RS can react as an alimentary prebiotic for patients with T2DM. They have found that RS2 decreased HbA1c (−0.3%, −3.6%) and TNF-α (−3.4 pg/mL, −18.9%) significantly compared with the placebo group (p < 0.05). However, changes in FBS, hs-CRP and IL-6 were not significant in the RS2 group compared with the control group. In contrast to the previous finding by Heianza and co-workers (6), both studies done by Lotfollahi and Gargari recorded decreased HbA1c levels after the intervention (31, 32).

Although RS2 can improve the glycemic status and inflammatory markers in some individuals with T2DM, more studies are needed to confirm the efficacy of RS2 as an adjunct therapy in diabetes, as reported by Gargari et al. (32). Similarly, Alfa and colleagues conducted another study to evaluate the tolerability, glucose, and insulin modulating ability in healthy MID and ELD adults upon consumption of *MSPrebiotic*^®^ digestion resistant starch (DRS) (33). *MSPrebiotic*^®^ is a product made from unmodified natural DRS from potatoes and contains an active ingredient, *Solanum tuberosum* extract. The subjects were randomized and required to consume 30 g/d of either *MSPrebiotic*^®^ or placebo for 12 wks. Several parameters including blood glucose, lipid profile, CRP, TNF-α, IL-10, and insulin resistance (IR) were measured over the whole study period. Baseline data from this study indicated that the ELD population had a significantly higher elevated glucose and TNF-α compared to MID adults. Interestingly, a significant difference was observed in blood glucose, insulin levels and HOMA-IR in ELD subjects who consumed *MSPrebiotic*^®^ compared to placebo. Unfortunately, there were no significant changes found in MID subjects. A newly published systematic review to evaluate the effect of prebiotics on metabolic and inflammatory biomarkers in T2DM patients found that ~70% of the study reported improvements in glycemia including HBA1c, HOMA-IR and inflammatory markers (64).

Besides its action on diabetic parameters and gut microbiota, RS2 was also found to affect macronutrient oxidation based on a study conducted in healthy adults aged 25–45 yrs in 2019. The subjects who consumed RS and RS high fiber (RSF) demonstrated a 32% increase in fat oxidation (*p* < 0.05) with a concomitant 18% decrease in CHO oxidation (*p* < 0.05). Furthermore, insulin responses were unaltered after breakfast but lower in RS and RSF after lunch, at equivalent glucose concentrations. Based on this finding, RS and RSF were expected to improve insulin sensitivity at later meals, though there has been no earlier expression of insulin response (34). Nevertheless, this study was carried out on healthy adults and dissimilar findings can be documented if the trial was performed on patients with T2DM.

**Figure 3:**
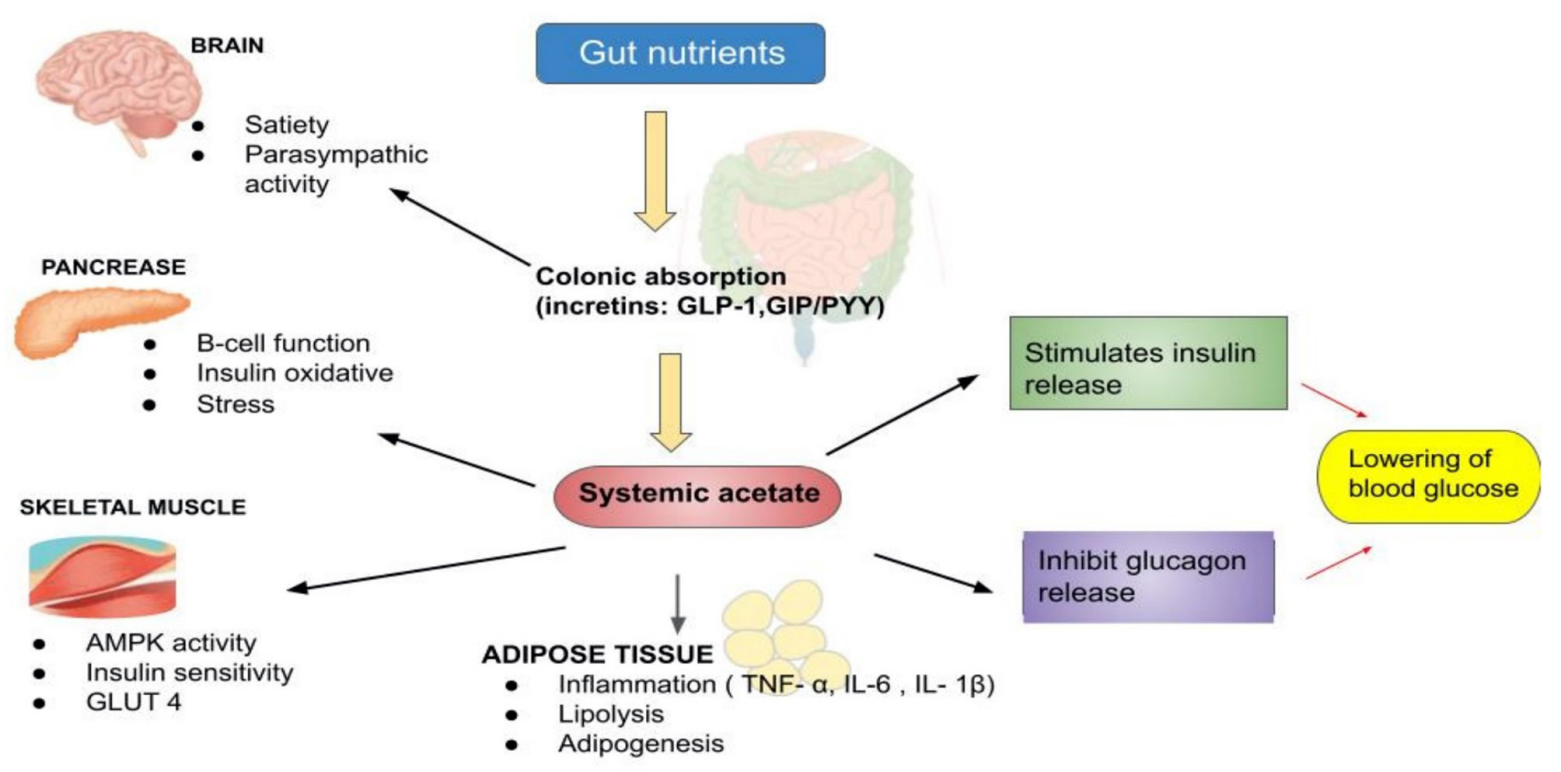
The schematic actions of gut nutrients on insulin release [Adapted from: (65, 66)].

Based on our search, we also noticed that RS3-based interventions had been extensively investigated. In 2019, Belobrajdic and his colleagues (35) had conducted a crossover-controlled study on 20 healthy non-diabetic men who consumed bread made from HAW and LAW. They determined that flour processing did not affect the glycemic, insulinemic or increatin response. However, HAW bread had lower insulinemic and increatin (24–30% less) than LAW (35). In other words, replacing LAW with HAW flour is more practical as an effective strategy for lowering glycemic and insulinemic responses in healthy men. Figure 3 represents the schematic action of gut nutrients on insulin release (65, 66). From a mechanistic perspective, vast data suggest that acetate has an important regulatory role in body weight control and insulin sensitivity via lipid metabolism and glucose homeostasis.

In another study, healthy subjects who consumed EAW bread showed significantly lower PPG response than those who consumed the reference white wheat bread during the first 120 min (*p* < 0.05), although there were no differences in insulin responses (36). This novel wheat with 38% EAW was field-grown under standard cultural practices in the experimental station of the University of Tuscia (Viterbo, Central Italy). Interestingly, higher RS content in bread significantly reduced the GI (*p* < 0.001). Despite promising results, this study was limited in terms of sample size that does not guarantee effectiveness in a larger population. Hence, further investigations are warranted for a more effective outcome on the roles of EAW in the improvement of postprandial glycemic response (36).

Besides using wheat bread, the effect of grain sorghum flour was also investigated in both healthy and pre-diabetic subjects, respectively (37, 38). Grain sorghum flour contained 22.7 ± 0.8, 43.8 ± 0.8, and 33.5 ± 0.1, whereas wheat flour contained 37.5 ± 0.3, 47.4 ± 0.4, and 15.1 ± 0.1 of RDS, SDS, and RS; respectively. In healthy subjects, incremental changes in plasma glucose and plasma insulin concentrations were recorded with the grain sorghum muffin treatment. Furthermore, the glucose responses were significantly lower, particularly at 45–120 min intervals (*p* < 0.05) and mean insulin responses reduced at 15–90 min intervals compared to control (*p* < 0.05). This study demonstrated a significant reduction at about 26% in the mean glucose iAUC responses with sorghum at 2,871 ± 163 mg compared to wheat at 3,863 ± 443 mg (~3 h)/dL. Similarly, insulin responses also significantly decreased from 3,029 ± 965 μU (~3 h)/L for wheat to 1,357 ± 204 with sorghum (*p* < 0.05), respectively. Furthermore, significant reduction on postprandial blood glucose and insulin responses within 45–120 min intervals (*p* < 0.05) along with reduction in glucose iAUC by 35.0%, from 5457.5 ± 645.4 to 3550.0 ± 428.9 mg (~3 h)/ dL (*p* < 0.05) were recorded in pre-diabetic men. Additionally, the mean insulin iAUC was also significantly lowered by 36.7%, from 7254.6 ± 1228.9 to 4589.3 ± 737.2 mU (~3 h)/L (*p* < 0.05). In short, both studies documented interesting results that grain sorghum is an excellent functional ingredient to assist in managing glucose and insulin levels among healthy individuals and controlling blood glucose and insulin levels among the pre-diabetic population (37, 38).

A group of researchers has also embarked on a study to evaluate the effect of a new formula, PPB-R-203 (a retrograded starch product manufactured by Pharma Power Biotec Co., Ltd.) vs. commercially available food on blood glucose regulation and safety in both healthy and T2DM individuals aged 20–65 yrs old (39). The findings demonstrated that mean blood glucose concentration and glucose iAUC were lower with the PPB-R-203 regimen than control among T2DM patients. In brief, PPB-R-203-based food managed to improve postprandial hyperglycemia in T2DM patients without giving a risk of hypoglycemia compared with commercially available food (39).

Recently, a comparison study has been done by Sanders and his friends to assess the metabolic effects in subjects that consumed 300 g of cooked then chilled potatoes containing ~18 g of RS compared to a control diet matched for calories and macronutrients but with <1 g of RS over a 24 h period (40). By introducing RS-enriched potatoes, the values for muscle insulin sensitivity index (MISI), HOMA2 insulin sensitivity (%S), and HOMA2 β-cell function (%B) increased, although the differences did not approach statistical significance compared with the control group. However, a significant reduction of the FPG (*p* < 0.05) was recorded with a slight insignificant decrease of fasting plasma insulin (*p* = 0.077). In addition, breath hydrogen at the end of 300 min was significantly higher (*p* < 0.05), and plasma FFA was significantly lower (*p* < 0.05) in the potato condition compared to the control. Even though RS-enriched potatoes may have a favorable impact on CHO metabolism, additional research with a large study sample is necessary.

Newly developed high fiber white rice (HFWR) has also been investigated for its GI and effect on glycemic parameters among healthy volunteers. The subjects were given HFWR and commercial white rice (WR) diets containing 50 g of available CHO as the test foods. HFWR was documented with a 23% lower GI value than WR, in which HFWR was found to be of medium GI (61.3) whereas WR was of higher GI (79.2). The dietary fiber, RS, and amylose content were also significantly higher in HFWR than WR. These results showed that the newly developed HFWR had a lower GI and other favorable characteristics than WR. Hence, the HFWR could help reduce the overall dietary GI and the glycemic load (41). However, it may be necessary to conduct an additional study to identify the HFWR capacity on its impact on blood glucose parameters, particularly for those with disabilities in glucose management.

Another valuable product that has been recognized as one of the ancient cultivated cereal grains globally is barley (*Hordeum vulgare* L.). Barley is an enriched source of high fiber contents, at about 15.6 g/100 g (67). Nomura and his colleagues conducted a study to determine the GI, blood glucose, postprandial blood glucose and iAUC after BARLEYmax^®^ consumption in healthy Japanese subjects (42). BARLEYmax^®^ is a unique, non-genetically modified barley developed by Australia's Commonwealth Scientific and Industrial Research Organization (CSIRO) (68). They found that the GI of the BARLEYmax^®^ was 24.3, and glucose iAUC was statistically lower than those who took the ordinary glucose drink. They presumed that the presence of fructans in BARLEYmax^®^ at 11% could contribute to glycemic response reduction, which is higher than other components comprised of RS, β-glucan and arabinoxylan at 3, 6 and 7%, respectively (42).

Meanwhile, Zhu and his colleagues have investigated the potential benefits of a combination of several non-cereal starchy (LS, AD and dried LB) compared with (MI, BR and AB) into a glycemic management diet while taking the pre-soaking and cooking duration into account (43). Unfortunately, despite being whole grains, the waxy and pre-soaked cereals can be very high-GI food. Moreover, pulses such as the AB can maintain a low GI even after a long-time pre-soaking and prolonged cooking and the LS is a valuable low-GI food ingredient in staple food which behaved more like pulses in terms of GR, while the AD and LB were more like millet and strongly dependent on cooking practice for GI values. Thus, they concluded that careful choice of whole-grain materials, minimized pre-soaking, and moderate cooking may be critical for successful glycemic management for people with impaired glucose metabolism. On the contrary, Yulianto and his friends evaluated the content of RS and GI of Cr-PR coated with herbal extracts (cinnamon bark powder, pandan leaf and bay leaf) with concentrations of Cr-PR at 1, 2, and 3%, respectively (44). In their study, 18 non-diabetic individuals were required to consume one type of cooked rice sample equivalent to 50 g of glucose. They recorded that the RS content of Cr-PR coated with herbal extracts ranged between 8.27 and 8.84% and the highest RS was gained from coating rice with 3% cinnamon extract. On the whole, it was classified into the low GI category as it ranged from 29 to 40 and the lowest GI was observed in Cr-PR coated with 6–9% of cinnamon extract (29–30) (44).

So far, no specific high-fiber or CHO-rich foods are arguably superior to the others. Sandberg and his colleagues have conducted a randomized, controlled, crossover overnight study to investigate the effect of WG-based products on glucose and appetite regulation (45). In this noteworthy study, four rye-based evening test meals were given to 21 healthy subjects consisting of either whole-grain RFB or a 1:1 ratio of RFB/RKB, with or without added RS, while WWB was used as the reference meal. Interestingly, the findings showed that the evening test meal of RFB/RKB + RS resulted in decreased postprandial glucose and insulin responses and increased the gut hormone in plasma the following morning after the standardized breakfast. Apart from that, consumption of RFB and RFB/RKB also resulted in a decreased feeling of hunger in the test subjects (AUC 0–210 min), while all the rye-based evening meals resulted in reduced fasting FFA and increased breath hydrogen concentration. This finding suggests that WG bread had the potential to improve cardiometabolic variables at 11 to 14.5 h perspective in healthy humans (45).

Research on cardiometabolic risk markers that consumed RB has also been evaluated in 38 healthy individuals aged 52–70 yrs. Insulin sensitivity index, PYY levels and plasma acetate fasting concentrations, butyrate, and total SCFA were recorded to increase with RB + RS2 bread product consumption. In addition, the insulin sensitivity index was found to correlate positively with working memory test performance (Figure 3). Intriguingly, IL−1β fasting levels were found to decrease after the intervention (46).

Besides RS2- and RS3-based diets studied by numerous researchers, wheat-based RS4-enriched flour (30% v/v) has also been investigated in 86 subjects above 18 yrs old (47). Except for TG, the RS4-enriched flour significantly reduced cholesterol levels (TC, HDL and non-HDL, including LDL). Although mean blood glucose levels were lowered with RS4 consumption, the differences did not approach statistical significance compared to the control. Mesa Garcia and her colleagues have taken a step ahead of research to study a new fructose-free DSF in fragile elderly T2DM patients for 6 wks and evaluate the influence of total enteral feeding on the glycemic control and diabetes-derived cardiovascular risk biomarkers (48). These new fructose-free DSF and RS4-enriched DSF formulations effectively controlled glycemic by decreasing blood HbA1c, and some improvements were documented in MCP-1, sE-selectin, and sVCAM-1 and PAI-1 plasma levels. While this formulation managed to improve glycemic control and cardiovascular risk biomarkers, lipid metabolism has not changed, which is helpful for the nutritional treatment of frail elderly T2DM patients.

Apart from RS4- enriched flour and DSF formulations, two fermentable-fibers were also investigated on postprandial SCFA and second-meal glycemic response among healthy OWO vs. LN participants (NCT02562014) (49). Intriguingly, no significant differences were observed in postprandial SCFA responses between LN and OWO participants after acute fiber consumption. They reported that any effect of the colonic fermentation of dietary fiber on glycemic responses was not attributed to SCFA, as IN increased SCFA serum without reducing the second-meal glycemic response. The low glucose response after RS consumption at 4–6 h is consistent with other studies that find the RS therapy increases insulin susceptibility and reduces insulin and C-peptide responses (49). The decreased glycemic reaction is probably not related to SCFA but may be related to the decrease in FFA recovery. A higher-than-expected amount of available but SDS in the RS ingredient used would have explained the acute reduction of FFA, as both prolonged and enhanced absorption of CHO managed to reduce postprandial FFA rebound. Based on these findings, they predicted that IN is fermented promptly in the colon and that breath-hydrogen and serum SCFA concentrations are rapidly rising, while RS is fermented gradually for 6 h after consumption with far smaller increases of breath hydrogen and serum SCFA. Moreover, the results did not demonstrate the impact on glucose metabolism of the acute increments in SCFA. In addition, the results did not support the hypothesis that colonial SCFA was increased due to obesity/overweight conditions (49).

## Conclusion

In conclusion, our review highlighted the benefits of RS that are loaded with antidiabetic properties. A considerable amount of RS consumption acts differently through several pathways to reduce the severity and exert better control of the diabetic condition. Although a specific RS type could not be highlighted for a better action mechanism, we observed that both RS2 and RS3 were among the most extensively explored form of RS over the last decade. The search results demonstrated that most of the studies were focused on insulin secretion and plasma glucose. Nevertheless, the RS effectiveness on HbA1c, inflammatory markers and lipid profile were also investigated in several studies. However, a proper comparison and conclusion are deemed inappropriate in view that the study parameters varied in terms of study duration, sample size, subjects and their metabolic conditions, intervention doses, and the intervention base products. Furthermore, the underlying mechanisms involved in the action of RS for diabetes control should be explored in more detailed perspectives for a better understanding.

## Author Contributions

All authors listed have made a substantial, direct, and intellectual contribution to the work and approved it for publication.

## Conflict of Interest

The authors declare that the research was conducted in the absence of any commercial or financial relationships that could be construed as a potential conflict of interest.

## Publisher's Note

All claims expressed in this article are solely those of the authors and do not necessarily represent those of their affiliated organizations, or those of the publisher, the editors and the reviewers. Any product that may be evaluated in this article, or claim that may be made by its manufacturer, is not guaranteed or endorsed by the publisher.

## Abbreviations

Aβ: Amyloid-beta
AD: Adzuki bean
ACh: Acetylcholine
AChE: Acetylcholinesterase
AD: Alzheimer's disease
AFM: Atomic Force Microscope
BMI: Body mass index
BR: Waxy black rice
CHO: Carbohydrate
CRP: C-reactive protein
CSIRO: Commonwealth Scientific and Industrial Research Organization
ELD: Elderly
FFA: Free Fatty acid
FPG: Fasting Plasma Glucose
FSIVGTT: Frequently sampled intravenous glucose tolerance test
GI: Glycemic index
GLP1: Glucagon-like peptide-1
IFG: Impaired fasting glucose
IGT: Impaired glucose tolerance
iAUC: Incremental area under the curve
IR: Insulin resistance
IL: Interleukin
IN: Inulin
HbA1c: Glycated hemoglobin
HAM: High-amylose maize
HAW: High-amylose wheat
HDL: High-density lipoprotein
HFWR: High fiber white rice
hs: High-sensitive
LAW: Low-amylose wheat
LB: Lily bulb
LDL: Low-density lipoprotein
LN: Lean
LS: Lotus seed
MCP-1: Monocyte chemotactic protein-1
MI: Millet
MID: Mid-age
MISI: Muscle insulin sensitivity index
MTT: Meal tolerance test
NEFA: Non-esterified fatty acids
OGTT: Oral glucose tolerance test
OWO: Overweight or obese
PAI-1: Plasminogen activator inhibitor-1
PYY: Peptide YY
RB: Rye-based bread
RDS: Rapidly digestible starch
RFB: Rye flour bread
RKB: Rye kernels bread
RS: Resistant starch
SCFA: Serum short-chain FA
SDS: slowly digestible starch
TC: Total cholesterol
TEE: Total energy expenditure
TG: Triglycerides
T2DM: Type 2 diabetes
sVCAM-1: Soluble vascular cell adhesion molecule −1
WG: Whole grain rye
WR: White rice
WWB: Wheat flour bread
2hPG: 2-h plasma glucose.
